# Molecular mechanisms underlying the ferroptosis-induced epileptiform activity in mouse cortical slices

**DOI:** 10.3389/fncel.2026.1758230

**Published:** 2026-02-25

**Authors:** Sara Petrillo, Federica Loia, Michela Giustizieri, Caterina Torda, Sara Cairoli, Bianca Goffredo, Marina Trivisano, Luca de Palma, Federico Vigevano, Nicola Specchio, Enrico Cherubini, Fiorella Piemonte

**Affiliations:** 1 Unit of Muscular and Neurodegenerative Diseases, Bambino Gesù Children’s Hospital, IRCCS, Rome, Italy; 2 European Brain Research Institute (EBRI)-Rita Levi-Montalcini Foundation, Rome, Italy; 3 Research Unit of Metabolic Diseases, Bambino Gesù Children’s Hospital, IRCCS, Rome, Italy; 4 Unit of Neurology, Epilepsy and Movement Disorders, Bambino Gesù Children’s Hospital, IRCCS, Full Member of European Reference Network on Rare and Complex Epilepsies EpiCARE, Rome, Italy; 5 Department of Developmental Disabilities, IRCCS San Raffaele, Rome, Italy; 6 University Hospitals KU, Leuven, Belgium

**Keywords:** 15-LOX, epilepsy, ferroptosis, GPx4, RSL-3, system Xc^−^

## Abstract

**Introduction:**

Ferroptosis, a newly defined iron-dependent programmed cell death, characterized by excessive accumulation of lipid peroxides and reactive oxygen species, is involved in epilepsy, particularly in those forms resistant to drugs. In a previous study, we have demonstrated that exposure of mouse cortical slices to the ferroptosis inducer RSL-3, induces interictal epileptiform discharges. To investigate the mechanisms underlying ferroptosis-induced epileptic activity in RSL-3-treated cortical slices, we analysed the expression of the main contributors to ferroptosis susceptibility in cells.

**Methods:**

The expression of proteins involved in RSL-3 induced ferroptosis pathways were analysed on mouse cortical slices by Western blot and qRT-PCR. The epileptogenic response was investigated by electrophysiological patch clamp recordings, in current clamp mode, from layer 2/3 mouse cortical slices.

**Results:**

In cortical neurons, the ferroptosis induction by RSL-3 was associated with a reduced expression of the GPX4/GSH redox pathway, responsible for the clearance of lipid peroxides, and an upregulation of 15-LOX, which promotes the formation of lipid peroxides. Furthermore, the cysteine/glutamate antiporter Xc^−^, a modulator of excitotoxicity in the brain, was up-regulated either after RSL-3-treatment or by incubating neurons with 4-HNE, the bioactive product of lipid peroxidation. Interestingly, 4-HNE was able to generate spontaneous interictal bursts.

**Discussion:**

Our findings establish a direct link between lipid peroxidation and Xc^−^ activity. These results suggest that the xCT antiporter may represent a promising therapeutic target for treating ferroptosis-mediated drug-resistant epilepsy.

## 1 Introduction

Epilepsy is characterized by recurrent seizures that are driven by multifaceted mechanisms, including oxidative stress and neuro-inflammation (Manavi et al., 2025). The pathophysiology of epilepsy is multifactorial and involves a complex interplay between genetic predisposition, environmental factors, and cellular processes. Among them, oxidative stress and neuroinflammation have been hypothesized as mechanisms responsible for the initiation and progression of epileptogenesis (Ji et al., 2025; Stockwell et al., 2017).

In about 30% of patients, epileptic discharges are difficult to treat with conventional drugs, exhibiting a drug-resistant epilepsy (DRE). Growing evidence has demonstrated that ferroptosis, an newly defined iron-dependent programmed cell death, characterized by excessive accumulation of lipid peroxides and reactive oxygen species particularly within cell membranes (Stockwell et al., 2017), is involved in epileptogenesis particularly in patients with DRE (Huang et al., 2024; Xu et al., 2025). We recently found a marked dysregulation of three ferroptosis key markers in the blood of children with epilepsy: (i) the 4-hydroxy-2-nonenal (4-HNE), the main by-product of lipid peroxidation; (ii) glutathione (GSH), essential in the antioxidant response; (iii) the enzyme glutathione peroxidase 4 (GPX4), the primary mediator of lipid peroxides detoxification (Petrillo et al., 2021). Furthermore, we also demonstrated that, in mouse cortical slices, the well-known ferroptosis inducer RSL-3 can generate, in a concentration dependent way, interictal discharges, confirming the crucial role played by ferroptosis in epilepsy (Giustizieri et al., 2023).

The aim of this study is to investigate the mechanisms underlying ferroptosis-induced epileptic activity in RSL-3-treated mouse cortical slices. Particularly, we examined the main contributors to ferroptosis susceptibility in cells, including the enzymes 15-LOX, which plays a key role in catalyzing the formation of lipid peroxides, GPX4 that is crucial in counteracting ferroptosis by neutralizing lipid peroxides, and GSH, the primary ROS scavenger and cofactor for the GPX4 activity. In addition, we analysed, in mouse cortical slices, the expression of the glutamate antiporter xCT/SCL7A11 (also known as system Xc^−^), which plays crucial role in ferroptosis and modulating excitotoxicity in several brain disorders (Dahlmanns et al., 2023). Overall, the ultimate goal of this study is to identify new therapeutic targets for ferroptosis-induced epileptic discharges, as possible alternatives to conventional treatments and surgical resection of the epileptic focus, which currently represents the only therapeutic options.

## 2 Methods

### 2.1 Animals and ethical approval

All experiments were performed on C57BL/6J mice, in accordance with the Italian Animal Welfare legislation (D. L. 26/2014), implemented by the European Committee Council Directive. Experiments were approved by local veterinary authorities, the EBRI ethical committee and the Italian Ministry of Health (protocol F8BBD. N. HCD). All efforts were made to minimize animal suffering and to reduce the number of animals used.

### 2.2 Cortical slices

Cortical slices were obtained from postnatal (P) day P30–P40 old animals (male and female), using a standard protocol (Schubert et al., 2001) and as reported in Giustizieri et al. (2023). Briefly, after being anesthetized with an intraperitoneal injection of a mixture of tiletamine/zolazepam (zoletyl, 80 mg/kg body weight) and xylazine (rompun, 10 mg/kg body weight), mice were decapitated. The brain was quickly removed from the skull and placed in ice-cold artificial cerebral-spinal fluid (ACSF) containing (in mM): sucrose 65, NaCl 85, glucose 10, KCl 2.5, NaH_2_PO_4_ 1.25, NaHCO_3_ 25, CaCl_2_ 0.5, MgCl_2_ 7, saturated with 95% O_2_ and 5% CO_2_. Coronal slices (300 mm thick) were cut with a vibratome and stored at room temperature (22–24 °C) in a holding bath containing the same solution as above. After incubation for at least 1 h, an individual slice was transferred to a submerged recording chamber and continuously perfused at 33–34 °C with oxygenated ACSF of the following composition (in mM): NaCl 130, glucose 10, KCl 3.5, NaH_2_PO_4_ 1.2, NaHCO_3_ 24, CaCl_2_ 3, MgCl_2_ 0.5, at a rate of 3 mL/min. For each experimental approach, data were collected from several slices (3–10) obtained at least from 3 different mice.

### 2.3 Electrophysiology

Electrophysiological recordings were obtained from layer 2/3 pyramidal neurons of the somato-sensory barrel cortex, visually identified with a 60 X water immersed objective, mounted on an upright Olympus CX23 microscope, equipped with differential interference contrast optics and an infrared video camera (Scientifica, UK). Membrane potential values were not corrected for liquid junction potentials. Whole cell patch clamp experiments were performed mainly in current-clamp configuration. Patch electrodes were pulled from borosilicate glass capillaries (WPI, Florida, USA). They had a resistance of 3–5 MΩ when filled with an intracellular solution containing (in mM): K-gluconate 135 mM, KCl_4_, NaCl_2_, HEPES 10 mM, EGTA 4 mM, MgATP 4 mM, NaGTP 2 mM; pH adjusted to 7.3 with KOH; 290 mOsm. Whole-cell current-clamp recordings were obtained using a Muticlamp 700B (Axon Instruments, Sunnyvale, CA, USA). The stability of the patch was checked by repetitively monitoring the input and series resistance during the experiments. Cells exhibiting >15% changes were excluded from the analysis.

### 2.4 Data analysis

Data were transferred to a computer hard disk after digitization with an A/D converter (Digidata 1550B, Molecular Devices). Data acquisition (digitized at 10 kHz and filtered at 1 kHz) was performed with pClamp 11.1 software (Molecular Devices, Sunnyvale, CA, USA). Input resistance and cells capacitance were measured online with the membrane test feature of the pClamp software.

### 2.5 Western blot analysis

Cortical slices were lysed on ice with RIPA buffer and protease inhibitors. An amount of 30 μg proteins was subjected to SDS PAGE on 4–12% denaturing gel and probed with the following antibodies: Gpx4 (1:1000, R&D Biotechne, Minneapolis, USA), 15-LOX (1:1000, Abcam, Bristol, UK), Gcl (1:6000, Proteintech, Rosemont, USA), xCT (1:1000, Abcam, Bristol, UK) and b-Actin (1:30,000, Sigma Aldrich) as loading control. Immunoreactive bands were detected using the Lite Ablot Extend Long Lasting Chemiluminescent substrate (Euroclone, Milan, Italy). Signals derived from appropriate HRP-conjugated secondary antibodies (Bethyl Laboratories, Montgomery, TX, United States) were captured by Chemi DocTM XRS 2015 (Bio-Rad Laboratories, Hercules, CA, United States) and densitometric analysis was performed using Image Lab software (Version 5.2.1, Bio- Rad Laboratories).

### 2.6 GSH assay

Glutathione levels were detected in mice cortical slices by an enzymatic re-cycling assay. Samples were de-proteinized with 5% (w/v) sulphosalycilic acid (SSA, Sigma-Aldrich, St. Louis, MO, USA) and the glutathione content was determined after dilution of the acid soluble fraction in Na-phosphate buffer containing EDTA (pH 7.5) with the ThioStar^®^ glutathione detection reagent (Arbor Assays, Michigan, MI, USA), using GSH as standard (Sigma Chemicals, St. Louis, MO, USA). The fluorescence was measured by an EnSpire^®^ Multimode Plate Reader (Perkin Elmer, Waltham, MA, USA). Protein concentration (mg/ml) was detected by the BCA method (ThermoFisher, Walthman, MA, USA) and GSH levels were expressed as nmol/mg prot.

### 2.7 Cysteine assay

The mice cortical slices were washed twice in PBS, sonicated 10″ for 3 times and centrifuged at 1000 × g for 5 min. Cell lysates were precipitated with 75 μL of 10% 5-sulfosalicylic acid (SSA), left for 1 h at 4 °C and centrifuged at 20000 × g for 15 min. Supernatant (25 μL) was analysed by reverse-phase high-performance liquid chromatography and fluorescence detection (HPLC-FLD). The HPLC-system was an Agilent Technologies 1,260 Series equipped with a fluorescence detector operating at Excitation wavelength 385 nm and Emission wavelength 515 nm. Data obtained were analysed with the ChemStation program (Agilent Technologies Deutschland GmbH, Waldbronn, Germany). Method validation was performed based on the US Food and Drug Administration (FDA) guideline for industry bioanalytical method validation and European Medicines Agency (EMA) guideline. The validation of the assay was performed including selectivity, specificity, linearity and limit of quantification, accuracy and precision, matrix effects and recovery, and stability. Protein concentration (mg/ml) was detected by the BCA method (ThermoFisher, Walthman, MA, USA) and cysteine levels were expressed as nmol/mg prot.

### 2.8 Blood sample collection

Blood samples from four children with epilepsy were collected into EDTA Vacutainer Tubes (Becton Dickinson, Rutherford, NY) and leukocytes were isolated by adding 10% dextran. After 45 min at room temperature, the upper phase was centrifuged at 2600 × g (5 min) and the pellet washed with 0.9% NaCl and stored at −20 °C until RNA extraction. Blood from five healthy children, without history or clinical evidence of neurological, neuropsychological, oncological, and inflammatory diseases, was collected at the Department of Laboratory Medicine of the Bambino Gesù Children’s Hospital during routine blood tests and used as controls. All participants signed an informed consent, and the study was approved by the Ethics Committee of Bambino Gesù Children Hospital in Rome.

### 2.9 Quantitative real-time PCR (qRT-PCR)

Total RNA was extracted from leukocytes using Total RNA Purification Plus Kit (Norgen, Biotek Corp., Thorold, ON, Canada), according to manufacturer’s protocol. RNA quantification was performed on a NanoDrop2000 Spectrophotometer (Thermo Scientific, Waltham, MA, USA). The purity of RNA was assessed by measuring the ratio of absorbance at 260 nm and 280 nm. An amount of 1 μg RNA was reverse transcribed with the SuperScriptTM First-Strand Synthesis system and random hexamers as primers (Life Technologies, Carlsbad, CA, USA). The expression levels of SLC7A11 were measured by qRT-PCR in an ABI PRISM 7500 Sequence Detection System (Life Technologies) using TaqMan Master Mix (ThermoFisher Scientific, Walthman, MA, USA). Data were analyzed using the 2^∆∆Ct^ method with TBP (TATA box binding protein) as housekeeping gene and expressed as fold change relative to controls.

### 2.10 Statistical analysis

Statistical analysis was performed using the GraphPad Prism 5.0 Software (San Diego, CA, United States). Statistically significant differences between two groups were analyzed using Student’s *t*-test. All data are shown as mean ± SD. Statistical significance was defined as *p < 0.05, **p < 0.01, ***p < 0.001 compared to untreated samples.

## 3 Results

### 3.1 The treatment with the ferroptosis inducer RSL-3 decreases GPX4 protein levels and up-regulates 15-LOX in mouse cortical slice

**Figure 1:**
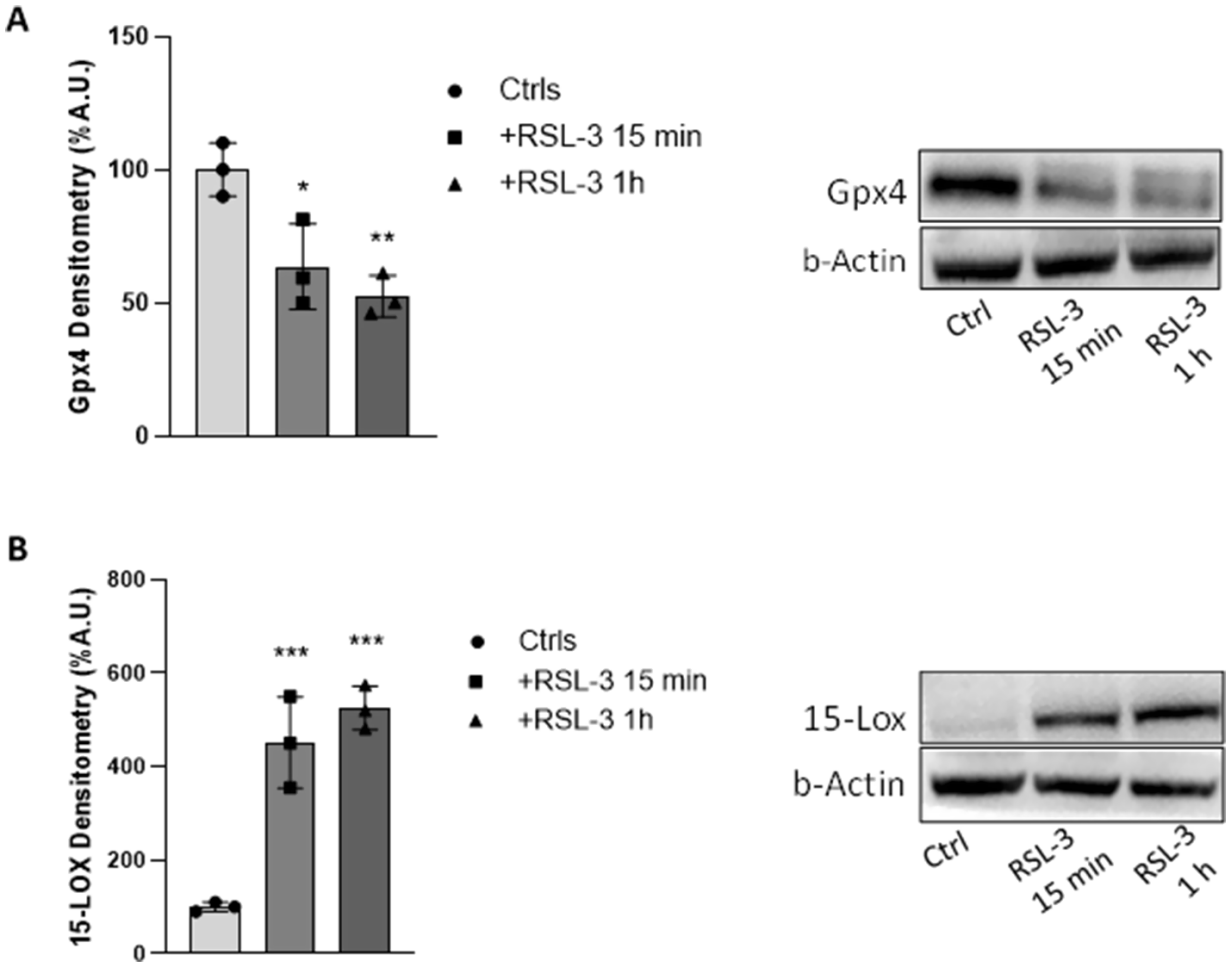
RSL-3 decreases GPX4 protein levels and up-regulates 15-LOX in mouse cortical slice. Densitometric analysis (left) and representative western blot (right) of GPX4 **(A)** and 15-LOX **(B)** protein levels in mouse cortical brain slices before and after 15 min and 1 h treatment with RSL-3 (5 µM). Experiments were conducted in triplicates and values expressed as mean ± SD. *p < 0.05, **p < 0.01, ***p < 0.001 as compared to controls (Ctrls).

It is well known that RSL-3 specifically acts by inhibiting the activity of GPX4, an antioxidant enzyme that protects cells against membrane lipid peroxidation (Li et al., 2021). Thus, to validate the effect of the drug, we first analysed the expression of this enzyme in RSL-3-treated cortical slices. As expected, western blot analysis revealed a significant reduction of GPX4 expression in RSL-3-treated cortical slices, if compared with untreated ones (Figure 1A). A 36 and 47% reduction was found, respectively, after 15 min and 1 h exposure to RSL-3 (5 μM), confirming previous evidence of GPX4 degradation because of RSL-3 interaction. The enzyme 15-lipoxygenase (15-LOX), which contributes to the formation of lipid peroxides by catalysing the oxygen incorporation into carbon-hydrogen bonds of polyunsaturated fatty acids (PUFAs) (Yang et al., 2016; Shintoku et al., 2017), was consistently up-regulated after 15 min (4.5-fold) and 1 h (5.3-fold) RSL-3 administration (Figure 1B). Therefore, in cortical neurons, ferroptosis generates an imbalance between GPX4 and 15-LOX, with GPX4 no longer ensuring cell protection against lipid peroxidation and 15-LOX strongly contributing to the increase of lipid oxidation.

### 3.2 The RSL-3-induced ferroptosis reduces GSH levels in mouse cortical slices

**Figure 2:**
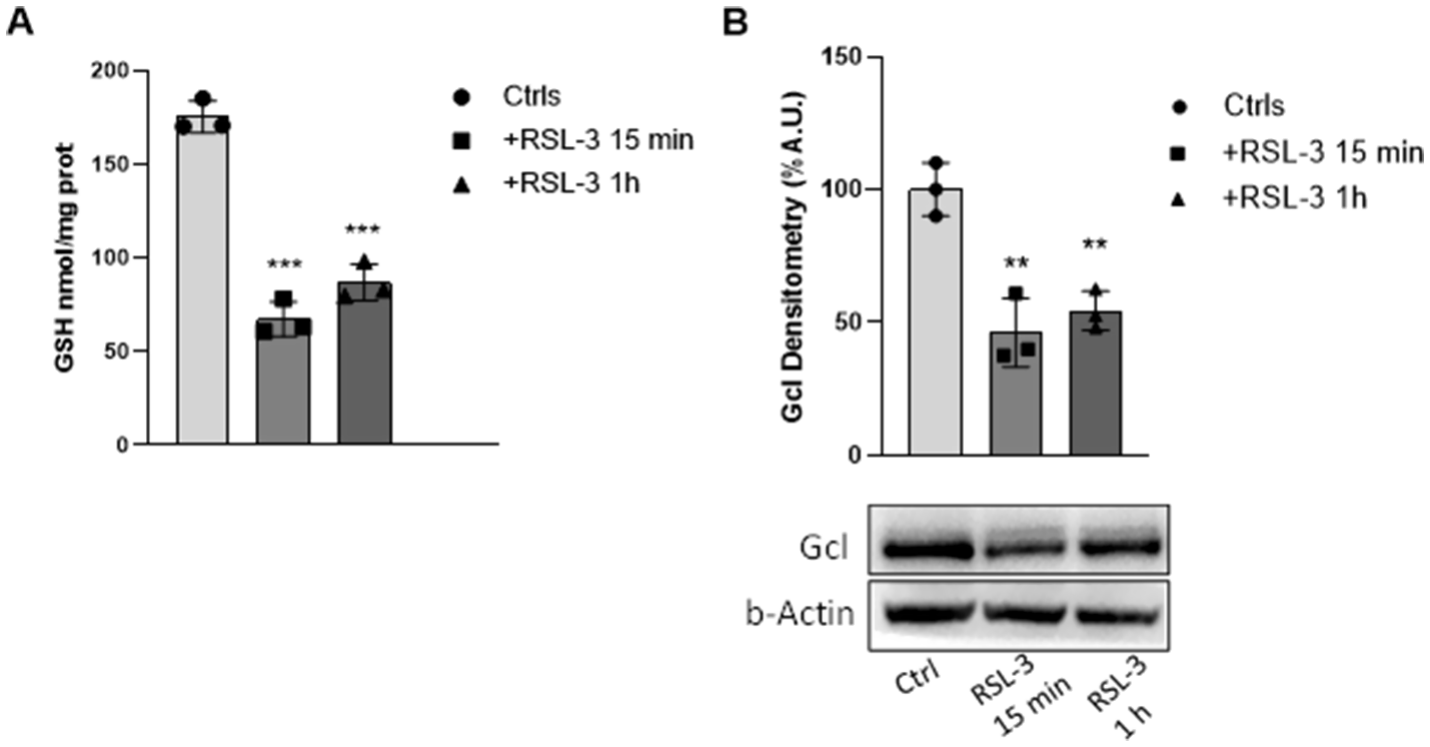
RSL-3 reduces GSH levels in mouse cortical slices. Glutathione (GSH) levels **(A)** and Gcl protein expression **(B)** as determined in mouse cortical slices before and after 15 min and 1 h RSL-3 treatments (5µM). Values represent mean ± SD of three independent experiments. **p < 0.01, ***p < 0.001, compared with controls’ group (Ctrl).

Along with the GPX4/15-LOX imbalance, another important hallmark of ferroptosis is represented by the GSH content, for its essential role as a direct scavenger of ROS and as a cofactor for the GPX4 activity itself. As shown in Figure 2A, in mouse cortical slices, the GSH concentration was consistently decreased to 64 and 52% at 15 min and 1 h after RSL-3-treatment, respectively, thus deeply reducing the antioxidant protection of the brain. Furthermore, as the GSH deficiency may be caused not only by its increased consumption in the antioxidant protection, but also by a reduced *de novo* synthesis, we analysed the expression of the GSH synthesis rate-limiting enzyme, *γ*-glutamyl-cysteine ligase (GCL). In RSL-3-treated mouse cortical slices, a decrease of GCL protein content of 54 % and 45% was found at the two times of drug application, respectively (Figure 2B).

### 3.3 The RSL-3-induced ferroptosis decreases cysteine levels in mouse cortical slices

**Figure 3:**
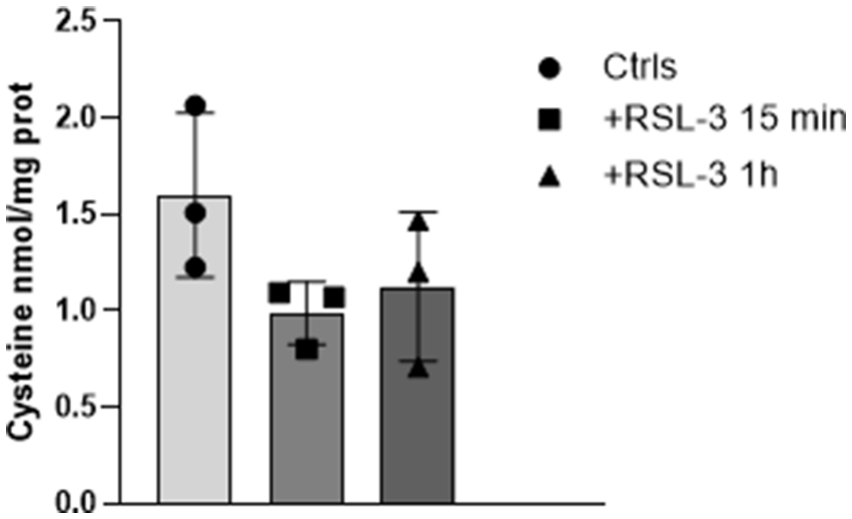
RSL-3 decreases cysteine levels in mouse cortical slices. Cysteine levels in mouse brain cortical slices before and after 15 min and 1 h treatment with RSL-3 (5 µM). Values represent mean ± SD of three independent experiments.

The GSH synthesis strongly depends on the availability of cysteine, one of the three aminoacids composing the tripeptide. To understand if the GSH synthesis could be affected by the cysteine neuronal concentration, we analysed by HPLC the cysteine levels in cortical slices undergoing RSL-3-mediated ferroptosis. Interestingly, we found 41% and 32% cysteine decreases after, respectively, 15 min and 1 h RSL-3 treatment (Figure 3). Although these values were not significantly different, the reduced expression of cysteine may contribute to slow down GSH synthesis, further weakening the antioxidant defence system of the brain.

### 3.4 The Xc^–^system is up-regulated in cortical slices undergoing RSL-3 induced ferroptosis

**Figure 4:**
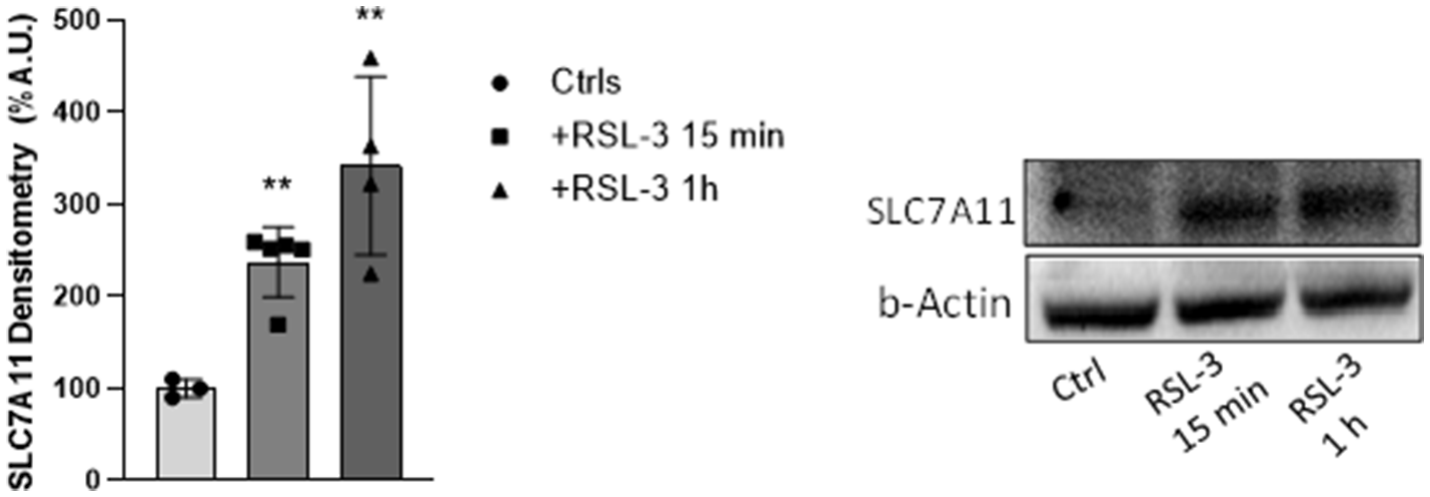
The Xc- system is up-regulated in cortical slices undergoing RSL-3 induced ferroptosis. Densitometric analysis (left) and representative western blot (right) of SLC7A11 protein levels in mouse cortical brain slices before and after 15 min and 1 h treatments with RSL-3 (5 µM). Experiments were conducted in triplicates and values expressed as mean ± SD. **p < 0.01, as compared to controls’ group (Ctrl).

The intracellular concentration of cysteine is largely dependent on the activity of the antiporter Xc^−^, which exchanges one molecule of glutamate outside the cell for one cysteine inside. This homeostasis is critical in central nervous system (CNS) to maintain balanced extracellular glutamate levels, to prevent excitotoxic effects, and to ensure adequate levels of cysteine for the GSH synthesis and the intracellular antioxidant response. To evaluate if low levels of cysteine in cortical slices could be due to a deficient expression of Xc^−^, we analysed SLC7A11, the catalytic subunit of system Xc^−^, in RSL-3 treated samples of cortical slices. Surprisingly, we found a significant 2.4- and 3.4-fold increase of the protein amount after 15 min and 1 h treatment, respectively (Figure 4), possibly reflecting a compensatory brain response to the GSH and cysteine decreases by the Xc^−^activation. Overall, these data provide evidence that the cysteine/GSH/GPX4 defence axis, responsible for the clearance of lipid peroxides, is down-regulated in cortical neurons undergoing ferroptosis-mediated epileptic activity, whereas 15-LOX, which promotes the formation of lipid peroxides, and Xc^−^, the modulator of excitotoxicity in brain, are up-regulated.

### 3.5 Could 4-HNE be the link between ferroptosis and epileptic activity in mouse cortical slices?

**Figure 5:**
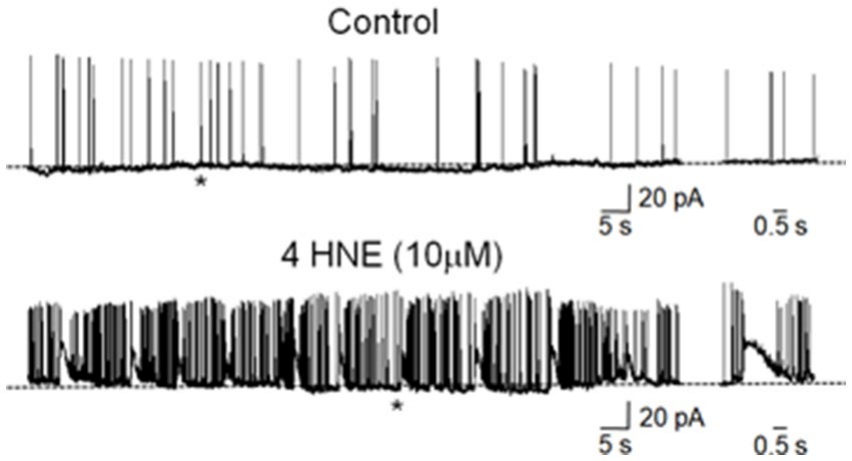
4-HNE induced an enhancement of spontaneous synaptic activity in mouse cortical slices. Whole cell patch clamp recordings (-70mV) in current clamp configuration from a layer 2/3 pyramidal neuron in the mouse somatosensory cortex before (upper trace) and after 15 min bath application of 4-HNE (10µM, lower trace). On the right spontaneous firing and burst activity (indicated by the asterisks), recorded on an expanded time scale.

Based on our findings showing an imbalance between 15-LOX-catalyzed lipid peroxidation and GPX4-mediated lipid peroxides detoxification, we wondered if the main endogenous by-product of lipid peroxidation, 4-HNE, could elicit an epileptogenic response comparable to that promoted by RSL-3 in mouse cortical slices. We therefore performed electrophysiological patch clamp recordings, in current clamp mode, from layer 2/3 mouse cortical slices, before and after exposure for 15 min to 4-HNE (10 μM). As illustrated in Figure 5, the application of 4-HNE in the bath induced, within 15 min, an enhancement of spontaneous synaptic activity in cortical slices and the appearance of epileptogenic interictal bursts in all respects similar to those induced by RSL-3 (Giustizieri et al., 2023), that persisted after washing out of the drug. A burst was defined as a transient sequence of two or more action potentials occurring at high frequency (>20 Hz) (Zucca et al., 2017), often driven by a slow depolarizing after potential which allowed recruiting additional action potentials. These data demonstrate that the main by-product of lipid peroxidation can induce epileptogenic discharges in mouse cortical slices, thus leading to suggest that 4-HNE is a possible mediator between ferroptosis and epileptogenesis.

### 3.6 4-HNE has a modulatory effect on 15-LOX and Xc^−^ comparable to that elicited by RSL-3 in mouse cortical slices

**Figure 6:**
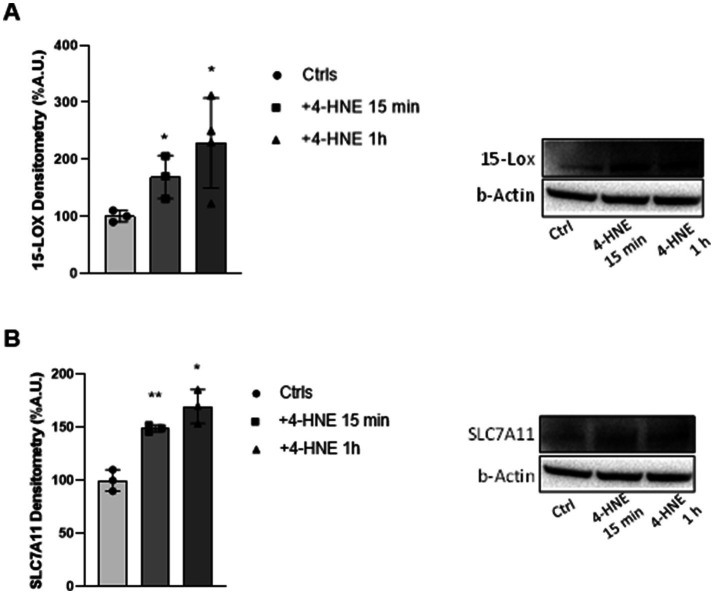
4-HNE has a modulatory effect on 15-LOX and Xc-system in mouse cortical slices. Densitometric analysis (left) and representative western blot (right) of 15-LOX **(A)** and SLC7A11/ Xc- **(B)** protein levels in mouse cortical brain slices before and after 15 min and 1 h treatment with 4-HNE (10 µM). Experiments were conducted in triplicates and values expressed as mean ± SD. *^p^ < 0.05, **^p^ < 0.01, as compared to control (Ctrl).

Having demonstrated the epileptogenic effect of 4-HNE on cortical slices, we wondered if, as in the case of RSL-3, 15-LOX and Xc^−^ could be affected by the treatment with 4-HNE. As reported in Figure 6, Western blot analyses showed a significant up-regulation of both 15-LOX (1.7-fold at 15 min; 2.2-fold at 1 h, Figure 6A) and Xc^−^ (1.5-fold at 15 min; 1.7-fold at 1 h, Figure 6B) after 4-HNE treatment, thus supporting a central role for these proteins in the 4-HNE-induced epileptic activity. Overall, our findings strengthen the ferroptosis as crucial contributor to the pathophysiology of epilepsy and open the way to the identification of new disease targets, with 4-HNE as a link between ferroptosis and epileptogenesis in mouse cortical slices.

### 3.7 The Xc^–^system is up-regulated in leukocytes of children with epilepsy

**Figure 7:**
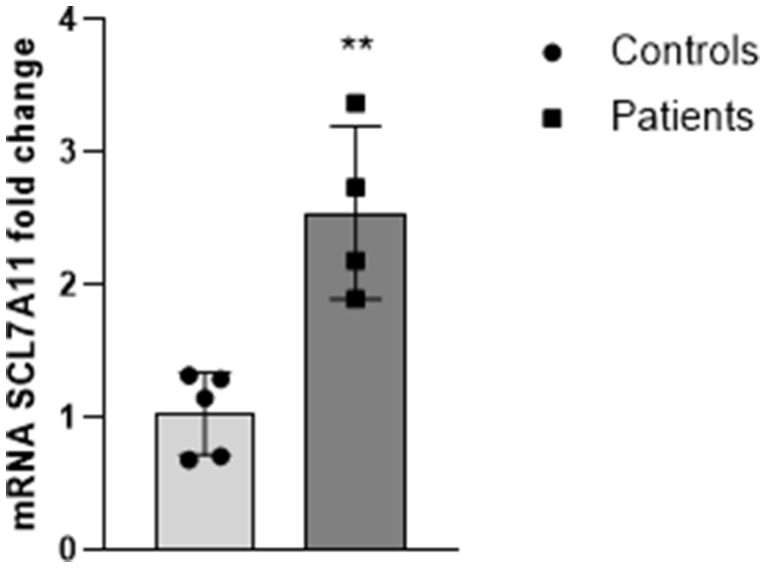
The Xc- system is up-regulated in leukocytes of children with epilepsy. Real-time PCR analysis of SLC7A11/ Xc- in leukocytes of 4 pediatric patients with epilepsy. Values are expressed as mean ± SD. Statistical significance is defined as ** ^p^ < 0.01, respect to healthy subjects (Controls).

Moving from these data, and from our previous study highlighting a significant increase of 4-HNE levels in plasma of children with epilepsy (Petrillo et al., 2021), we decided to evaluate the Xc^−^ expression in the blood of a little cohort of our patients, to understand if the system responsible for the Excitatory/Inhibitory balance in the brain could be modulated also in patients, such as in ferroptosis-induced mouse cortical slices. As reported in Figure 7, Xc^−^ is over-expressed in blood of 4 pediatric patients with epilepsy, thus providing evidence for this protein as a potential peripheral biomarker in epilepsy, able to mirror pathological changes occurring in the brain.

## 4 Discussion

In order to contribute to the development of drugs that could be alternatives to surgical resection, currently the only therapeutic option in some drug-resistant epilepsies, in this study we investigated the molecular mechanisms responsible for ferroptosis-dependent interictal discharges in mouse cortical neurons treated with RSL-3. Ferroptosis is an iron-dependent form of regulated cell death, due to the accumulation of lipid peroxides resulting from impairment of the glutathione peroxidase 4 (GPX4)/GSH redox pathway and/or activation of LOX-catalysed lipid peroxidation (Stockwell et al., 2017). Ferroptosis occurs in several neurological disorders (Xu et al., 2023; Mohan et al., 2021; Alatawi et al., 2025; Xu et al., 2024). In epilepsy, ferroptosis has been involved in the patho-physiological progression of the disease, and emerging studies demonstrated that the pharmacological inhibition of ferroptosis can mitigate neuronal damage (Huang et al., 2024; Xu et al., 2025; Jin et al., 2023; Su et al., 2024; Liu and Chen, 2022). Hallmarks of ferroptosis have been detected in several animal models of epilepsy (Kahn-Kirby et al., 2019; Mao et al., 2019; Du et al., 2022; Wang et al., 2022), but to date the sequence of molecular events underlying ferroptosis in epilepsy is still poorly understood and need further investigations.

Susceptibility to epileptic seizures can be caused by an Excitatory/Inhibitory imbalance in selective neural circuits, associated with multiple environmental and genetic factors underlying the different forms of the disease (Staley, 2015; Fiest et al., 2017). To discover new therapeutic targets for preventing seizures and protecting against multi-morbidities, such as cognitive dysfunction, full knowledge of the consequential signaling cascades beyond epileptogenesis is needed, especially in drug-resistant forms (Fisher et al., 2014; Fabisiak and Patel, 2022). Among the multiple actors involved in ferroptosis, in this study we focused on 15-LOX and GPX4 for their crucial and contrasting role in lipid peroxidation, the main trigger of the process (Yang et al., 2016; Seiler et al., 2008; Yang and Stockwell, 2016; Zhang et al., 2025). Indeed, under physiological conditions, GPX4 prevents ferroptosis by catalysing the neutralization of phospholipid peroxides into phospholipid alcohols (Seiler et al., 2008; Ursini et al., 1982; Ursini et al., 1985; Liang et al., 2022), whereas 15-LOX promotes lipid peroxidation by incorporating oxygen into carbon-hydrogen bonds of PUFA (Li et al., 2018; Mashima and Okuyama, 2015; Calder, 2005; Sadeghian and Jabbari, 2016). Therefore, the imbalance between the two enzymes can destabilize the lipid physiological asset and cause accumulation of oxidized lipids, which are the precursors of a reactions cascade ultimately leading to ferroptosis (Kahn-Kirby et al., 2019; Yang and Stockwell, 2016; Colakoglu et al., 2018). In a previous paper, we provided evidence that a partial inactivation of GPX4 activity occurs in the blood of children with epilepsy, an effect associated with a significant decrease of GSH levels and a consistent increase of the main by-product of lipid peroxidation (4-HNE) Petrillo et al., 2021). Thus, based on this information and on the observation that the ferroptosis inducer RSL-3 is able to trigger interictal epileptiform activity in mouse cortical neurons (Giustizieri et al., 2023) we investigated in depth the molecular mechanisms underlying ferroptosis-induced epileptogenesis in mouse cortical slices. Following RSL-3 application, we found that the GPX4/GSH redox pathway, responsible for the clearance of lipid peroxides, was decreased in cortical neurons, whereas 15-LOX, which promotes the formation of lipid peroxides, was up-regulated. This GPX4/GSH depletion not only weakens antioxidant defenses in cortical neurons, prompting cells to ferroptosis, but it can also contribute to triggering a vicious circle between oxidative stress and neuro-inflammation that is emerging as a critical player in the pathophysiology of epilepsy (Fabisiak and Patel, 2022). The up-regulation of 15-LOX further contributes to exacerbate neuronal ferroptosis, as also demonstrated by Zhang et al. (2022) in hippocampal neurons of epileptic rats where the inhibition of 15-LOX led to a significant reduction of neuronal ferroptosis and seizures. In this complex system, where all the factors must be in balance with each other, even cysteine plays an important role, being essential for the synthesis of GSH. Cysteine levels, indeed, are finely regulated inside cells and this regulation is mediated by the system Xc-, a chloride-dependent transporter responsible for the uptake of extracellular cysteine in exchange of the extrusion of one molecule of glutamate (Lo et al., 2008; Lewerenz et al., 2013). Therefore, a proper Xc^−^ function is necessary for maintaining the Excitatory/Inhibitory balance in the brain, to prevent excitotoxic states, and to ensure a correct synaptic transmission. To note, in diseases such as glioma, tumor-associated epilepsy, Alzheimer’s disease, Parkinson’s disease, amyotrophic lateral sclerosis or multiple sclerosis such regulation is lost (Dahlmanns et al., 2023). In patients with malignant glioma, in particular, the up-regulation of Xc^−^ has been found to be associated with seizures (Robert et al., 2015). Our findings show that Xc^−^ expression was significantly increased in RSL-3-treated cortical slices. However, this increase was insufficient to re-establish a correct cysteine content inside the tissue. Although indirectly, this finding supports our previous study showing an increase of glutamate release in RSL-3- induced ferroptosis (Giustizieri et al., 2023) and highlights the Xc^−^ antiporter as a potential new target for epilepsy. Xc^−^ was already proposed by Leclercq et al. (2019) as an important epileptic target in several rodent models of chronic epilepsy, where its genetic deletion led to decreased hyperexcitability and lower propensity to develop seizures. Authors also evidenced a significant Xc^−^ overexpression in hippocampal tissue from epileptic patients and in pilocarpine-treated mice, concluding that pharmacological inhibition of Xc^−^ can delay epileptogenesis and protect against seizures in several acquired forms of epilepsies. 15-LOX is another key enzyme responsible for the increase of cell susceptibility to ferroptosis, because of its important role in catalysing lipid peroxides formation (Hinman et al., 2018). 4-HNE is the most representative bioactive lipid peroxidation product. It can induce oxidative stress by itself, thereby amplifying the process of lipid peroxidation (Mattson, 1998; Dalleau et al., 2013; Schaur et al., 2015). In addition, 4-HNE has been shown to impair the glutamate transport in cortical astrocytes and in synaptosomes, suggesting 4-HNE as an important mediator for synaptosomal dysfunction and neuronal excitotoxicity (Keller et al., 1997; Blanc et al., 1998). In light of those previous evidences, we wonder if 4-HNE could play a role in epilepsy, possibly as epileptogenic mediator. Therefore, we performed electro-physiological recording of 4-HNE-treated mouse cortical slices and we evidenced, within 15 min after drug application, the appearance of spontaneous interictal discharges in all similar to those induced by RSL-3. We also found that 4-HNE induced a significant up-regulation of Xc^−^ in mouse cortical slices, suggesting a direct link between lipid peroxidation and Xc^−^ activity, and supporting the antiporter as a central pharmacological target for this disease. To note, we found a significant over-expression of Xc^−^ in the blood of a little cohort of children with epilepsy, thus providing a preliminary evidence for a new possible peripheral biomarker in epilepsy.

The induction of epileptiform discharges by 4-HNE in our *in vitro* model likely reflects a multifaceted disruption of neuronal excitability. Indeed, while 4-HNE is primarily known as a marker of oxidative stress, recent evidence highlights its role as a direct modulator of ion channel kinetics. It has been shown to influence the pathophysiology of epilepsy by modulating acid-sensing ion channels (ASICs) and inhibiting ferroptotic pathways (Shi et al., 2024). Furthermore, the covalent modification of TRPA1 and other redox-sensitive channels by 4-HNE can lead to sustained inward currents, promoting the transition from normal firing to paroxysmal activity (Milkovic et al., 2023). These channel-specific effects, combined with the documented inhibition of Na^+^/K^+^-ATPase and glutamate re-uptake (Siems et al., 1996; Keller et al., 1997), created a highly permissive environment for the generation and propagation of seizure-like events. Therefore, the 4-HNE-mediated ion channel dysfunction can represent a key driver of network hyper-excitability.

Another important aspect to consider is the involvement of the voltage-dependent anion channel (VDAC) on the RSL-3-induced ferroptosis that, on the contrary, is generally considered a process VDAC-independent. However, a role for VDAC in mitochondrial ferroptosis has been described by Oh et al. (2022), who proposed this channel as a link between the cytosolic and mitochondrial RSL3-mediated ferroptosis. Although still debated (Yang and Stockwell, 2008; Yang et al., 2020), with some papers excluding a role for VDAC in RSL3-induced ferroptosis (Yang and Stockwell, 2008) and others showing a modulation of VDAC2/3 by RSL-3 (Yang et al., 2020), however the contribution of the membrane channels in mitochondrial and cytosolic ferroptosis can be of great interest in epilepsy, especially to find new targeted therapies.

## 5 Conclusion

Ferroptosis plays a role in the development of epilepsy. Targeting ferroptosis-related molecules to treat epilepsy could be a feasible innovative approach. In particular, a combined therapy, including ASMs coupled with GPX4/GSH activating compounds and Xc^−^ regulators can be suggested for preventing seizures and protecting against cognitive dysfunction. However, how ferroptosis coordinates various processes in the pathophysiology of epilepsy is still not well understood and further in-depth studies are needed.

## Data Availability

The original contributions presented in the study are included in the article/Supplementary material, further inquiries can be directed to the corresponding author.
