# Supplementary material for: Molecular mechanisms underlying the ferroptosis-induced epileptiform activity in mouse cortical slices
Source: Front Cell Neurosci. 2026 Feb 25;20:1758230. doi: 10.3389/fncel.2026.1758230 (PMC12975598; doi:10.3389/fncel.2026.1758230)

Figure 1A GPX4

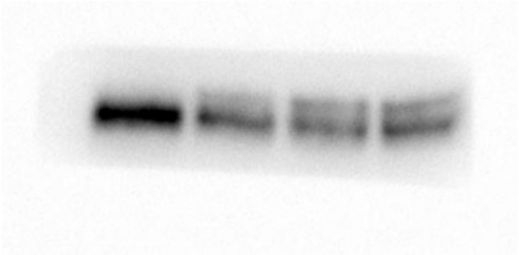

Figure 1A b-Actin

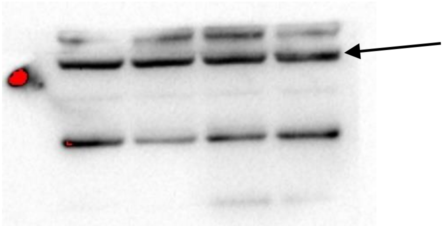

Figure 1 B 15-Lox

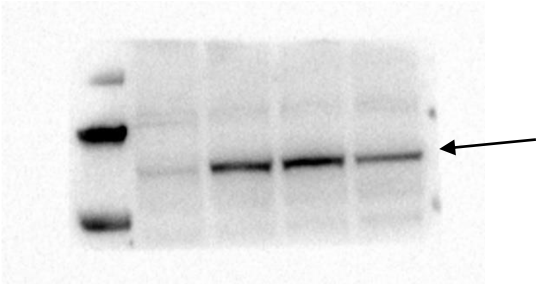

Figure 1 B b-Actin

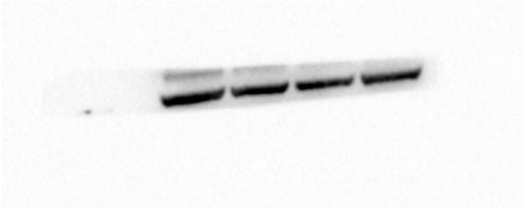

Figure 2B Gcl

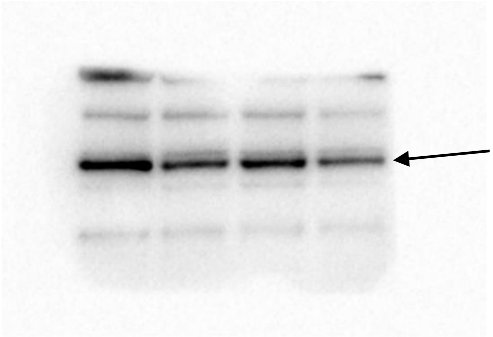

Figure 2B b-Actin

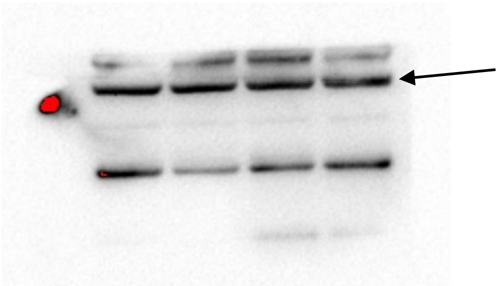

Figure 4 SLC7A11

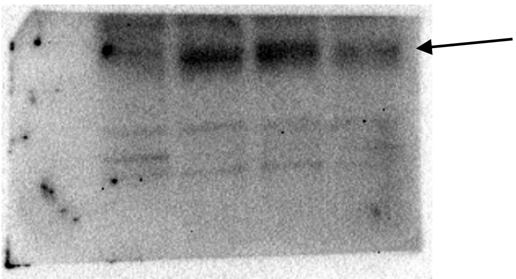

Figure 4 b-Actin

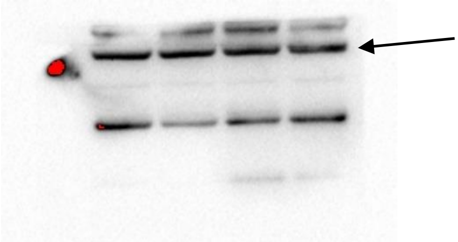

Figure 6A 15-LOX

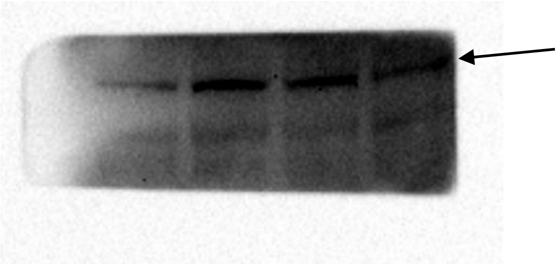

Figure 6 A b-Actin

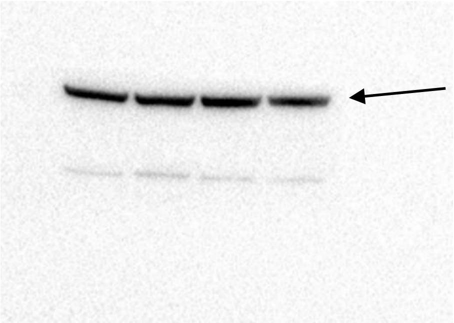

Figure 6 B SLC7A11

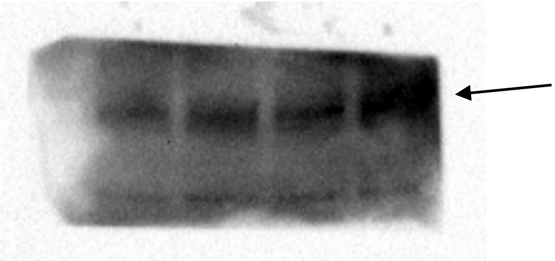

Figure 6 B b-Actin

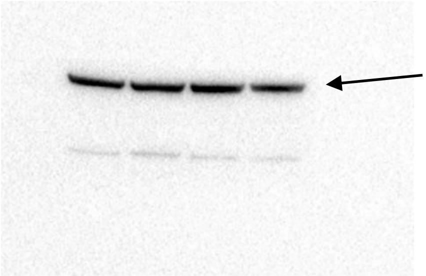

Supplement: Supplementary file 1 [file Data_Sheet_1.pdf]
